# Modes of death and clinical outcomes in adult patients with hypertrophic cardiomyopathy in Thailand

**DOI:** 10.1186/s12872-018-0984-0

**Published:** 2019-01-03

**Authors:** Nattakorn Songsirisuk, Veraprapas Kittipibul, Nilubon Methachittiphan, Vorawan Charoenattasil, Nath Zungsontiporn, Ittikorn Spanuchart, Saranya Buppajarntham, Charoen Mankongpaisarnrung, Sudarat Satitthummanid, Suphot Srimahachota, Pairoj Chattranukulchai, Smonporn Boonyaratavej Songmuang, Sarinya Puwanant

**Affiliations:** 1 Division of Cardiology, Department of Medicine, Faculty of Medicine, Chulalongkorn University, Rama IV Road, Bangkok, 10330 Thailand; 2 Cardiac Center, Thai Red Cross Society, King Chulalongkorn Memorial Hospital, Rama IV Road, Bangkok, 10330 Thailand

**Keywords:** Hypertrophic cardiomyopathy, Outcome, Echocardiography

## Abstract

**Background:**

There are limited data about modes of death and major adverse cardiovascular events (MACEs) in patients with hypertrophic cardiomyopathy (HCM) in South East Asian population. The aim of the study was to examine modes of death and clinical outcomes in Thai patients with HCM.

**Methods:**

Between January 1, 2009 and December 31, 2013, 166 consecutive patients with HCM diagnosed in our institution were evaluated. Five patients were excluded because of non-Thai ethnic groups (*n* = 3) and diagnosis of myocardial infarction at initial presentation documented by coronary angiography (*n* = 2). The final study population consisted of 161 patients with HCM. HCM-related deaths included: (1) sudden cardiac death (SCD) – death due to sudden cardiac arrest or unexpected sudden death; (2) heart failure – death due to refractory heart failure; or (3) stroke - death due to embolic stroke associated with atrial fibrillation. MACEs included: (1) SCD, sudden unexpected aborted cardiac arrest, fatal, or nonfatal ventricular arrhythmia (ventricular fibrillation or sustained ventricular tachycardia); (2) heart failure (fatal or non-fatal), or heart transplantation; or (3) stroke - fatal or non-fatal embolic stroke associated with atrial fibrillation.

**Results:**

One hundred and sixty-one Thai patients with HCM (age 66 ± 16 years, 58% female) were enrolled. Forty-two patients (26%) died over a median follow-up period of 6.8 years including 25 patients (16%) with HCM-related deaths (2%/year). The HCM-related deaths included: heart failure (52% of HCM-related deaths; *n* = 13), SCD (44% of HCM-related deaths; *n* = 11), and stroke (4% of HCM-related deaths, *n* = 1). The SCDs occurred in 6.8% of patients (1%/year). Eighty-four major MACEs occurred in 65 patients (41, 5%/year). The MACEs included: 40 heart failures in which 2 patients underwent heart transplants; 22 SCDs and nonfatal ventricular arrhythmias; and 22 fatal or nonfatal strokes.

**Conclusions:**

The most common mode of death in adult patients with HCM in Thailand was heart failure followed by SCD. About one-third of the patients experiencing heart failure died during the 6.8 years of follow-up. SCDs occurred in 7% of patients (1%/year), predominantly in the fourth decade or later.

## Background

Hypertrophic cardiomyopathy (HCM) is a common genetic disorder with heterogeneous phenotypic expression, clinical manifestation, and prognosis [1–3]. Recently, there has been a growing body of evidence about the natural history and prognosis of HCM, the data vary between unselected and selected patients from non-referral and referral centers [4–19]. The majority of those studies were derived from the United States and western countries [4–20]. However, little is known about modes of death and major adverse cardiovascular events (MACEs) in patients with HCM in South East Asian populations. The aim of this study was to examine the modes of death and MACEs in consecutive Thai patients with HCM.

## Methods

This study was conducted in a tertiary referral center. The study protocol was approved by the University Institutional Review Board (IRB).

### Participants

Between January 1, 2009 and December 31, 2013, 166 consecutive patients with HCM diagnosed in our institution were evaluated. Five patients were excluded because of non-Thai ethnic groups (*n* = 3) and diagnosis of myocardial infarction at initial presentation documented by coronary angiography (*n* = 2). The final study population consisted of 161 patients with HCM. The baseline characteristics and clinical data of the participants were obtained from medical records. Left ventricular hypertrophy (LVH) on electrocardiogram (ECG) was determined by Sokolow-Lyon criterion (S_V1_ + R_V5–6_ ≥ 35 mm). [21] When the heart rhythm was regular, heart rate was calculated by 1500 divided by the number of small (0.04 s) boxes between 2 successive R waves at the paper speed of 25 mm/second. When the heart rhythm was irregular, heart rate was calculated by the number(s) of QRS complexes in 10-s interval (i.e.50 large boxes) multiplied by 6. [22]. Patients with syncope, pre-syncope, or palpitation were assessed with holter monitoring. Exercise stress tests were performed in patients for risk stratification and functional capacity assessment. Patients were followed after being diagnosed with HCM until either events (HCM-related deaths, MACEs) occurred or contact with patients was lost.

### Echocardiography

An echocardiogram was performed in all patients using commercially available ultrasound machines, Vivid 7 GE-Vingmed (Milwaukee, WI) and IE-33 Philips (Philips Medical System, Andover, MA). The echocardiographic images were digitally stored in the EchoPAC and QLAB software package for off-line analysis. HCM was diagnosed based on echocardiographic evidence of left ventricular (LV) hypertrophy in the absence of other explainable causes of hypertrophy [23]. Patients with known mitochondrial disease, metabolic disease, malformation syndromes (e.g. Noonan syndrome), neuromuscular diseases (e.g. Friedreich’s ataxia), metabolic diseases (e.g. Anderson-Fabry, Pompe, Danon disease), or amyloidosis were excluded. The cardiac chamber quantification was acquired and measured according to the American Society of Echocardiography recommendation [24]. The maximal LV wall thickness was assessed by 2-dimensional echocardiogram. The presence of abnormal papillary muscle was based on morphology previously described [25]. The resting left ventricular outflow tract (LVOT) gradient was assessed and estimated by continuous wave Doppler under a resting physiologic condition [26]. Asymmetrical septal hypertrophy was defined as septal-to-free-wall ratio of ≥1.3 [15]. Apical HCM including pure and mixed apical HCM (apical/septal) was defined as previously described [23, 27]. Concentric phenotype of HCM was characterized by diffuse hypertrophy, LV wall thickness ≥ 15 mm or ≥ 13 mm in relatives, in the absence of other explainable causes of hypertrophy. Dilated HCM was defined as global LV systolic dysfunction of LVEF < 50% on index studies or during follow-up period in the presence of previous documentation which met the diagnostic criteria of HCM. Concomitant coronary artery disease was excluded either by coronary angiogram and or stress imaging study. [28–32].

### Clinical outcomes

The follow-up period was assessed from the initial evaluation that confirmed the diagnosis of HCM to the occurrence of death (HCM-related or non HCM-related) or MACE, or date of last contact. Death was determined by medical records, death certificates, or telephone reviews. MACE or the last contact date was assessed by medical records or telephone interviews.

HCM-related deaths included: (1) sudden cardiac death (SCD) – death due to sudden cardiac arrest or unexpected sudden death; (2) heart failure – death due to refractory heart failure; or (3) stroke - death due to embolic stroke associated with atrial fibrillation.

MACEs included: (1) SCD, sudden unexpected aborted cardiac arrest, fatal, or nonfatal ventricular arrhythmia (ventricular fibrillation or sustained ventricular tachycardia); (2) heart failure (fatal or non-fatal) which might have required heart transplantation; or (3) stroke - fatal or non-fatal embolic stroke associated with atrial fibrillation.

Heart failure was defined as symptomatic clinical syndrome, characterized by substantial functional limitation or exertional dyspnea (NYHA class ≥ III), orthopnea, paroxysmal nocturnal dyspnea, elevated jugular venous pulse, S3 gallop, rales, or edema, which may require hospitalization, especially if complicated by pulmonary edema.

Stroke was defined as transient or permanent neurological impairment and disability due to cardioembolic source, usually in the setting of atrial fibrillation. Fatal stroke was defined as death as a direct consequence of embolic stroke due to cardioembolic source, usually in the setting of atrial fibrillation.

### Statistical analysis

Frequency, percentage, median, and mean ± standard deviation (SD) were employed to express the data. The differences of variables were compared by a student’s t-test for variables with normal distribution and a Wilcoxon–rank sum test for variables with non-normal distribution. Categorical variables were compared using a Chi’s square test or Fischer exact test, where appropriate. Univariate Cox proportional hazards regression and multivariate Cox proportional hazards regression were employed to identify the predictors of HCM-related death and MACEs. Due to the small number of HCM-related deaths, the multivariate analysis was not performed. Kaplan-Meier curves were constructed to estimate survival or event-free survival between groups. Significant differences in survival or events were based on the log-rank test. *P* values < 0.05 were considered significant.

## Results

### Demographic and clinical characteristics

A total of 161 patients were enrolled in the study. The mean age was 66 ± 16 years, 58% were female. Of those, 8 patients were 35 years old or younger. The majority of patients resided in Bangkok or in the central region of Thailand. Twenty one patients (13%) were referred from other hospitals. Patients were followed for a mean period of 6.8 years. Baseline characteristics are shown in Table 1.

**Table 1:** Baseline characteristics of study patients

Variables	*n* = 161
Age (year)	66 ± 16
Age stratified by decade (years) [n (%)]	
– < 20 – 20-29 – 30-39 – 40-49 – 50-59 – 60-69 – 70-79 – 80-89 – ≥90	0 (0%) 4 (3%) 5 (3%) 19 (12%) 21 (13%) 36 (22%) 41 (25%) 32 (20%) 3 (2%)
Age at presentation (year)	58 ± 17
Female [n (%)]	93 (58%)
Family history of HCM/ SCD [n (%)]	17 (12%)/13 (9%)
History of syncope [n (%)]	8 (5%)
NYHA Class III-IV [n (%)]	21 (13%)
Systolic blood pressure (mmHg)	130 ± 18
Diastolic blood pressure (mmHg)	75 ± 12
Atrial fibrillation at presentation [n (%)]	12 (8%)
- Paroxysmal - Persistent/permanent	4 (3%) 8 (5%)
LVH on ECG [n (%)]	92 (57%)
Clinical presentation [n (%)]	
Asymptomatic with abnormal murmur	20 (12%)
Asymptomatic with abnormal EKG	27 (17%)
Dyspnea on exertion	40 (25%)
Palpitation	11 (7%)
Chest pain	33 (21%)
Syncope	8 (5%)
Heart failure	15 (9%)
Stroke	7 (4%)
Functional capacity in Metabolic Equivalents (METS)	7.9 ± 1.7
Hypotensive response to exercise [n (%)]	2 (1%)
Non-sustained ventricular tachycardia [n (%)]	2 (1%)
5-year ESC-SCD risk score	2.1 ± 0.6%
Type [n (%)]	
– Asymmetrical septal hypertrophy – Pure apical – Apical with mid LV involvement – Concentric – Localized – Pure Mid	81 (50%) 27 (17%) 24 (14%) 26 (16%) 2 (2%) 1 (1%)
Interventricular septal thickness (mm)	18 ± 6
Interventricular septal thickness ≥ 25 mm [n (%)]	24 (15%)
Interventricular septal thickness ≥ 30 mm [n (%)]	9 (5%)
Abnormal papillary muscle [n (%)]	36 (22%)
Resting systolic anterior motion [n (%)]	77 (48%)
Chordal Mild Moderate Severe	36 23 12 6
Significant LVOT gradient (> 30 mmHg) [n (%)]	38 (24%)
LVEDD (mm)	43 ± 8
LA diameter (mm)	40 ± 9
LAVI (ml/m2)	37 ± 18
LVEF (%)	74 ± 10
Dilated HCM (LVEF< 50%) [n (%)]	6 (4%)
Beta blocker [n (%)]	119 (74%)
Calcium channel blocker [n (%)]	19 (12%)
Amiodarone [n (%)]	14 (9%)
Anticoagulant [n (%)]	40 (25%)
Cardiac implantable electronic device [n (%)]	
– ICD – CRT-D – Pacemaker	5 (3%) 0 (0%) 8 (5%)

*CRT-D* implantable cardiac resynchronization therapy defibrillator, *ECG* electrocardiography, *HCM* hypertrophic cardiomyopathy, *ICD* implantable cardioverter defibrillator, *LA* Left atrium, *LAVI* left atrial volume index, *LVEDD* left ventricular end-diastolic diameter, *LVEF* left ventricular ejection fraction, *LVH* left ventricular hypertrophy, *LVOT* left ventricular outflow tract, *NYHA* New York Heart Association

### HCM –related deaths

During the follow-up period, there were 42 deaths (26% of patients) including 17 deaths unrelated to the HCM (e.g., malignancy, sepsis, gastrointestinal bleeding). HCM-related deaths occurred in 15.5% of patients (59.5% of the deaths, *n* = 25). HCM-related mortality was 2.3% per year. Heart failure was the most common mode of HCM-related death (8.0% of patients, *n* = 13), followed by SCD (6.8% of patients, *n* = 11), and stroke (0.6% of patients, *n* = 1).

***SCDs*** occurred in 11 patients. Of 11 patients with SCD, 4 died suddenly outside the hospital. Among those with out-of-hospital SCDs, 2 patients had history of exertion preceding SCD events. All 11 patients had left ventricular ejection fraction (LVEF) > 50%. The mean septal thickness was 19 ± 4 mm. One patient (9%) was on amiodarone. None of the patients with SCDs had implantable cardioverter defibrillator (ICD) prior to the events. The SCD mortality was 1.0%/year (*n* = 11).

***Heart failure-related deaths*** occurred in 13 patients. The median LVEF was 71% (32–88%). Two patients had LVEF < 50%. Six patients had an obstructive physiology with a resting LVOT gradient of ≥30 mmHg.

***Stroke-related death*** occurred in 1 patient who had atrial fibrillation with rapid ventricular response in the absence of anticoagulant therapy at the stroke onset.

#### Age distribution of the HCM-related deaths

**Fig. 1:**
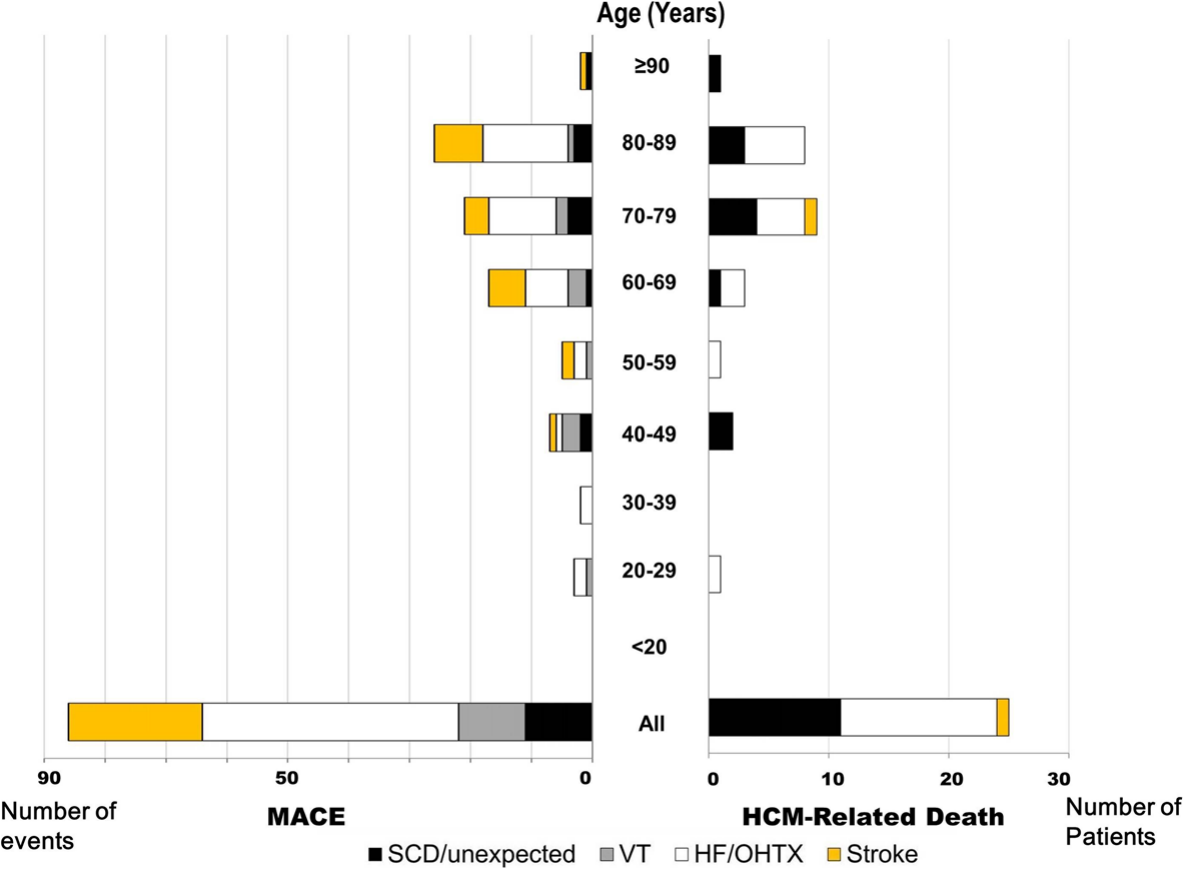
Relation of age distribution to mode of HCM-related death and to MACE, illustrated in number of patients

The mean age of HCM-related deaths was 72 ± 16 years (range of 27–90). Among the younger patients (35 years old or younger, *n* = 8), HCM-related death occurred in 1 patient (0.6% of patients). SCDs occurred in the fourth decade or later. No SCD occurred in patients ≤35 years of age (Fig. 1). Heart failure-related deaths occurred in a wide range of ages, 27–89 years, (Fig. 1). Stroke-related death occurred in one patient at the age of 76.

#### Relationships between the HCM-related deaths and clinical characteristics

There was no association between HCM-related deaths and their NYHA classes (*p* = 0.21); and major HCM phenotypes (chi square, *p* = 0.07). None of baseline medical therapy, ICD implants, or septal reductive treatment was a predictor of HCM-related death.

### Major adverse cardiovascular events (MACEs)

During the follow-up period, 65 patients had 84 MACEs that included: (1) fatal and nonfatal heart failure (40 patients); (2) SCDs and nonfatal ventricular arrhythmias (22 patients); and (3) fatal and nonfatal strokes (22 patients). Annual event rate of MACE was 5%.

#### SCD and nonfatal ventricular arrhythmia

SCD or nonfatal ventricular arrhythmia occurred in 22 patients (14% of patients). The age distribution of nonfatal ventricular arrhythmia extended to the younger age group (20–29 years), compared with that of SCD (> 40 years old) (Fig. 1).

#### Fatal and nonfatal heart failure

Fatal and nonfatal heart failure occurred in 40 patients (25% of patients) including 2 patients who underwent heart transplants. The age distribution of fatal and non-fatal heart failure was similar to that of heart failure–related death which occurred in a wide range of age (20–89 years old). Of 40 patients with heart failure, heart failure –related death occurred in 13 patients (33%). About one-third of patients experiencing heart failure died over 6.8 years.

#### Fatal and nonfatal embolic stroke

Fatal and nonfatal embolic strokes occurred in 22 patients (14% of patients), and were commonly found in older patients. Atrial fibrillation on ECG at initial presentation associated with fatal and nonfatal stroke, (Odd ratio = 4.06, 95% CI: 1.09–15.02, *p* = 0.04).

**Fig. 2:**
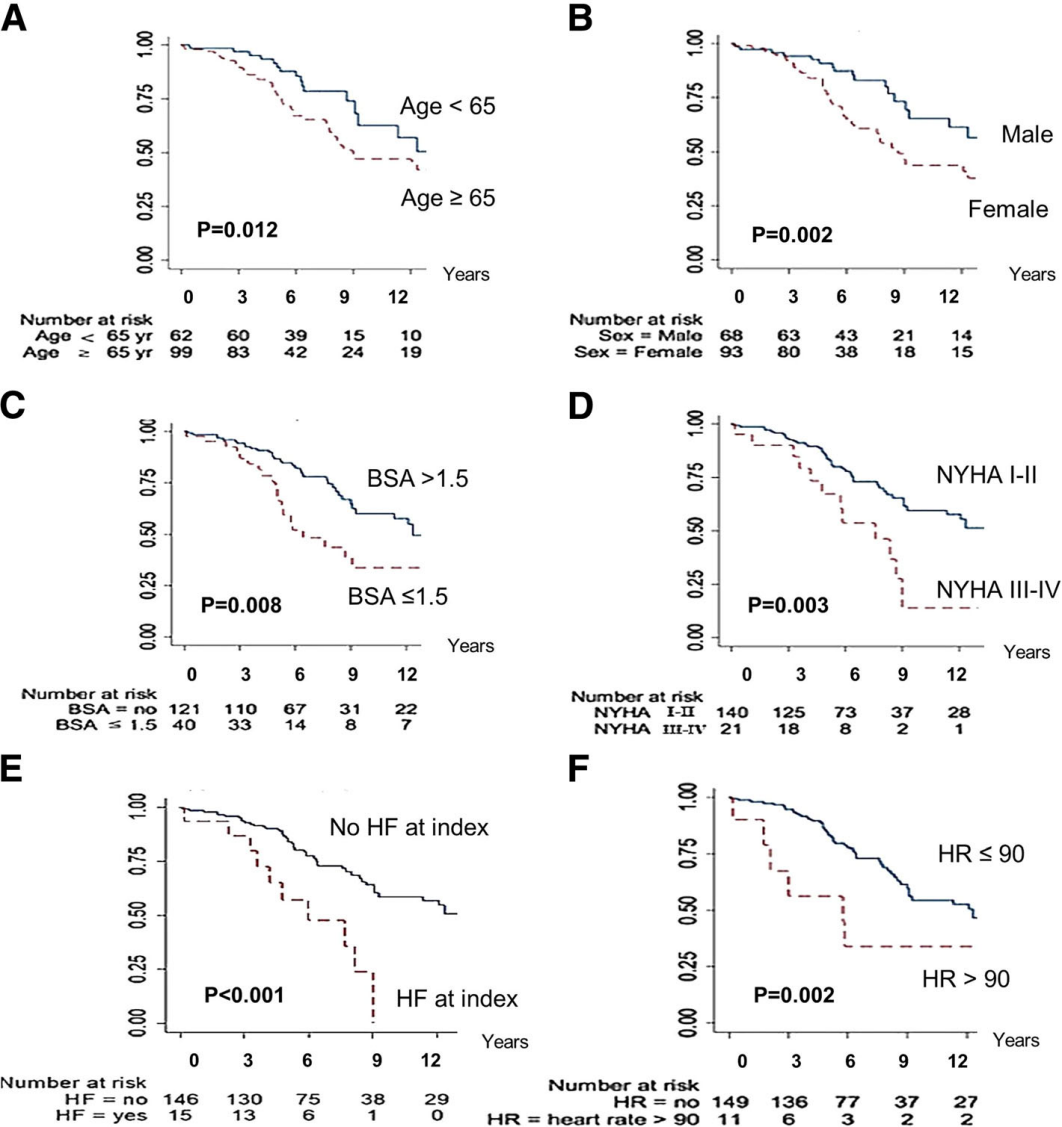
Kaplan–Meier curves for 6 variables for MACE- free survival BSA = body surface area; HR = heart rate; NYHA = New York Heart Association

By Cox proportional hazard analysis, there were 6 variables associated with MACEs: patients ≥65 years of age (HR = 2.01, 95% Cl: 1.15 to 3.5, *p* = 0.014), female gender (HR = 2.3, 95% Cl: 1.36 to 3.98, *p* = 0.002), a body surface area (BSA) ≤1.5 m^2^ (HR = 2.0, 95% Cl: 1.18 to 3.29, *p* = 0.009), NYHA III-IV (HR 2.6, 95% Cl: 1.36 to 4.97, *p* = 0.004), heart failure at presentation (HR 3.4, 95% Cl: 1.71 to 6.93, *p* = 0.001); and a heart rate > 90 bpm (HR = 3.0, 95% Cl: 1.18 to 3.29, *p* = 0.006) (Fig. 2a-f). Patients with atrial fibrillation on baseline ECG had a trend toward higher rate of MACEs (HR = 2.05, *p* = 0.05). Multivariate analysis revealed that a heart rate > 90 bpm (HR 2.18, 95% Cl: 1.10–4.86, p = 0.04), atrial fibrillation on baseline ECG (HR 2.81, 95% Cl: 1.30–6.06, *p* = 0.009), and heart failure at initial presentation (HR 2.93, 95% Cl: 1.23 to 6.97, *p* = 0.015) significantly associated with MACEs (Table 2). A heart rate > 90 bpm was detected more frequently in patients with heart failure at presentation (OR = 4.1, 95%CI: 1.17–14.12, *p* = 0.03).

**Table 2:** Predictors of HCM-related death and overall MACEs

Variables	HCM-Related Death		MACEs			
	Univariate Analysis		Univariate Analysis		Multivariate Analysis	
	Hazard Ratio	*p* Value	Hazard Ratio	*p* Value	Hazard Ratio	*p* Value
Age ≥ 65 years	0.78	0.66	2.01	0.01*	1.66	0.09
Female	1.24	0.65	2.33	< 0.01*	1.66	0.11
BSA ≤ 1.5 m2	2.03	0.11	1.97	0.01*	1.70	0.08
NYHA III-IV	1.36	0.58	3.00	< 0.01*	1.27	0.55
Heart rate > 90/min	2.56	0.07	3.05	< 0.01*	2.18	0.04**
Family History of SCD	0.88	0.73	1.03	0.94		
AF on ECG	1.83	0.28	2.05	0.05	2.81	0.01**
Heart failure at presentation	1.32	0.59	3.44	< 0.01*	2.93	< 0.01**
Maximal septal thickness ≥ 25 mm	1.15	0.80	0.97	0.94		
LVEF < 60%	0.60	0.36	1.75	0.12		
Septal reductive therapy	0.58	0.59	1.12	0.65		

*AF* atrial fibrillation, *BSA* body surface area, *ECG* electrocardiography, *LVEF* left ventricular ejection fraction; *MR* mitral regurgitation, *NYHA* New York Heart Association, *SCD* sudden cardiac death

### Septal reductive therapy

During the follow-up period, 10 patients (6%) and 1 (1%) patient underwent surgical septal myectomy and alcohol septal ablation, respectively. All of those had functional improvement after receiving septal reductive therapy.

### Cardiac implantable electronic device (CIED) therapy

Data of CIED at study enrollment was shown in Table 1. During the follow-up period, 9 (5%) and 2 patients (1%) underwent ICD and CRT-D implantation, respectively.

## Discussion

This is the first study that examines prognosis and clinical characteristics of adult patients with HCM in Thailand. This study was conducted in a tertiary referral center in Thailand and predominantly consisted of older adult patients. The major findings were: (1) overall deaths occurred in 26% of patients, 60% of deaths were HCM-related; (2) HCM-related deaths occurred in 16% of patients (2% /year); (3) SCDs occurred in 7% of patients (1%/year); (4) MACEs occurred in 41% of patients (5%/year); and (5) the most common mode of death in this study was heart failure followed by SCDs.

Although the patients in this study were enrolled in a tertiary care center, most patients (87%) were non-referral. The mean age of patients in the present study (66 ± 16 years) was older than that reported among other Asian populations [33] [34]. Notably, western cohorts reported that the mean ages were 30–45 years in HCM referral centers [4, 10, 11, 18–20] and 41–59 years in non-referral centers [14, 15, 17, 18, 35]. In the present study, pediatric and/or neonatal populations were not included. The percentage of patients with NYHA class III-IV in this study (12%) was consistent with that previously reported in HCM referral centers [4, 7, 16, 36].

### HCM phenotypes

Asymmetrical septal hypertrophy was the most common phenotype and was identified in half of patients in this study. Apical HCM was found in about one-third of patients. Yoshinaga et al. [37] reported a similar proportion among Japanese patients. The prevalence of apical phenotype varies between countries from 3 to 40%: Chinese (40%) [38], South Korean (24%) [23], Taiwanese (12%) [34]. These findings confirm that the apical HCM are not confined only in the Japanese population, but also found in other parts of Asia including Thailand. There was no association between the HCM phenotype and MACEs in the present study.

### HCM-related deaths

We found the rate of HCM-related death (16%), to be higher than that previously reported among American, Japanese, Taiwanese, and Chinese populations [18, 33–35, 38]. However, it was lower than that reported in community-based hospitalized patients by Seiler et al. (19%) [11] and Romeo et al. (22%) [10]. The vast majority of previous studies in non-referral centers described annual cardiovascular mortality rate in the rage of 0.6 to1.3% and up to 3.5% in HCM referral centers [4, 7–12, 35]. The annual HCM-related mortality rate in the present study was 2% which is consistent with that reported in Chinese patients with HCM [38]. The differences in HCM-related deaths and annual mortality rate among studies are attributed to the dissimilarity in study population, the treatment strategies, and the definitions of HCM-related death.

The most common mode of HCM-related death in this study was heart failure (52%), followed by SCD (44%), and stroke (4%). In contrast, Maron et al. [35] found that SCD was the most common mode of death which occurred in 51% of patients. As the SCD is more prevalent in younger patients, the older age group of the participants enrolled in this study could be a possible explanation of these findings. A wide range of age distribution of heart failure-related deaths in this study (27–89 years) was similar to that reported by Maron et al. [35].

### Sudden cardiac deaths (SCDs)

The annual SCD mortality rates in patients with HCM derived from community-based cohorts are ≤1%/year [12, 20]. Maron et al. [18] reported an annual mortality for SCD in unselected regional study populations was 0.7%. In the present study, annual mortality for SCD was 1%. This annual rate was consistent with that reported by Maki et al. [12]. The diversity of the annual SCD rates among studies was a result of the heterogeneity of study population, the ICD implant rate, the characteristics of patients, and the HCM phenotypes. The SCDs occurred in 7% of participants in this study, predominantly in the fourth decade or later. The absence of SCDs in younger patients could be explained by the small number of patients younger than 35 years of age (8 out of 161 patients) enrolled in the present study. Of those younger patients, one died as a consequence of heart failure with reduced ejection fraction, and one survived from a sustained ventricular tachycardia. The majority of patients who had SCDs (81%) were asymptomatic or mildly symptomatic preceding deaths. These findings were in accord with those previously published [4, 7, 35]. Maron et al. [35] also illustrated that 70% of the study patients had NYHA I-II preceding SCD. Family history of SCD was found in 9% of our patients which was similar to that in other studies [11, 13].

### Major cardiovascular adverse events (MACEs)

The MACEs occurred in 41% of the patients during the follow-up period of 6.8 years (5% per year). The MACE rate in the current study was much higher than that previously reported: 24% from Yoshinaga et al., Japan [37], 19% from Ho et al. [38], China, and 13% from Lee et al., Taiwan [34]. The diversity of MACE rates among studies may arise due to the heterogeneity of study population and different treatment strategies and the definition of MACEs. In the present study, we included death equivalent events (an appropriate ICD discharge, an intervention for ventricular arrhythmia or heart transplantation) as part of the definition of MACEs. Fatal and nonfatal embolic strokes in our study occurred more commonly in elderly patients. Similarly**,** Maron et al. [35] reported that strokes occurred more frequently in patients with more advanced age.

We found that a heart rate > 90 bpm, atrial fibrillation on baseline ECG, and heart failure at initial presentation were associated with HCM-related MACEs. A heart rate > 90 bpm was found more frequently in patients with heart failure as a consequence of sympathetic activation. On the other hand, a heart rate > 90 bpm or tachycardia resulting in impaired diastolic filling in a noncompliant ventricular chamber in HCM may lead to heart failure and adverse clinical events. Gwathmey et al. [39] demonstrated that tachycardia causes calcium overload leading to incomplete myocardial relaxation and a decrease in myocardial tension in HCM. Furthermore, approximately half of study patients with a heart rate > 90 bpm and heart failure symptoms did not receive beta-blocker at baseline. Beta-blocker therapy may affect heart rate reduction, symptom improvement, and major cardiovascular outcomes. Atrial fibrillation was substantially associated with stroke and cardiovascular events in patients with HCM [40]. Olivotto et al. [41] demonstrated that atrial fibrillation in patients with HCM significantly increased risk of stroke (OR = 17.7, *p* = 0.0001). Similarly, atrial fibrillation in the present study was an independent predictor of MACEs. These findings support that atrial fibrillation or tachycardia in patients with HCM is a significant marker for advanced stage, heart failure, or MACEs as a consequence of elevated left ventricular filling pressure, impaired left ventricular diastolic filling, loss of atrial contribution, and cardioembolic stroke. We found that heart failure at initial presentation was a predictor of MACEs. This is concordant with the report by Pasqualucci et al. [42], in which 5-year rate of adverse cardiac events in HCM patients with heart failure after development of NYHA class III-IV symptoms was significantly high (62%). Heart failure in HCM is associated with complex pathophysiology including arrhythmia, diastolic dysfunction, systolic anterior motion, left ventricular outflow tract obstruction, ischemia, or systolic dysfunction in advanced stage. The majority of patients in the present cohort had preserved ejection fraction. About a quarter and a half of patients had significant resting left ventricular outflow tract obstruction and substantial resting systolic anterior motion, respectively.

#### Study limitations

First, our study included a relatively small number of patients; however, this is the first and largest study of modes of death and clinical outcomes of adult HCM in Thailand and South East Asia. Second, the study population was partly contaminated by a referral bias as the present study was conducted in a tertiary referral center in Thailand. Third, autopsy was not performed in patients who suddenly died out of the hospital. Fourth, significant LVOT obstruction may be underestimated in the present study since LVOT gradient was measured under resting conditions. The number of patients with significant LVOT obstruction would have been higher if the gradient would have been routinely measured with provocative tests [43]. Lastly, this is a single tertiary center data which partly limits the generalizability. Further multi-center studies in patients with HCM in South East Asia are needed.

## Conclusions

The most common mode of death in adult patients with HCM in Thailand was heart failure followed by SCD. SCDs occurred in 7% of patients (1%/year) predominantly in the fourth decade or later.

## Abbreviations

EF: Ejection fraction
HCM: Hypertrophic cardiomyopathy
HR: Hazard ratio
ICD: Implantable cardioverter defibrillator
LV: Left ventricular
MACEs: Major adverse cardiovascular events
NYHA: New York Heart Association
SCD: Sudden cardiac death
